# Report of an Operation for Strangulated Femoral Hernia on a Patient Ninety-Five Years Old1Read at the thirty-eighth annual meeting of the Medical Association of Central New York, at Buffalo, October 24, 1905.

**Published:** 1906-04

**Authors:** Frederick H. Flaherty

**Affiliations:** Syracuse, N. Y.


### Report of an Operation for Strangulated Femoral Hernia On a Patient Ninety-five Years Old.[1]

BY FREDERICK H. FLAHERTY, M. D., Syracuse, N. Y.

I WISH briefly to report and thereby place on record a case of strangulated femoral hernia in a woman past 95 years of age, in which I operated, using local anesthesia, and who today is able to do her own housework for herself and son.

The case in brief is as follows: Mrs. R., 95 years old, born in Ireland, whose father and mother both lived to a ripe old age, while the patient herself has always been well, at least never suffered from any serious illness and never had but one child. Three years ago she was suddenly taken ill, complaining of much pain in the lower right abdominal quadrant. Her grandson, who is a physician, diagnosticated a right femoral hernia. With the aid of three other physicians he was unable to reduce the hernia by taxis.

---

1 Read at the thirty-eighth annual meeting of the Medical Association of Central New York, at Buffalo, October 24, 1905.

On account of her advanced age he did not consider any radical procedure justifiable; however, after 36 hours with the application of ice the hernia was reduced. A suitable truss was then fitted and she had relief from her trouble until July 30, of this year, at which time she again complained. After the third day she began to vomit large quantities of fecal smelling material, her pulse became extremely irregular, and it was apparent that she had a complete obstruction of the bowel. All efforts to reduce the hernia failed.

Because of her extremely advanced age, her irregular pulse and poor general condition I decided that it was a hopeless case, and that to give the patient a general anesthetic was out of the question; that she surely was dying, and so advised the family. I again saw the patient the next morning. Her pulse was even more irregular and she was still vomiting fecal matter. Upon consulting my colleague, Dr. D. M. Totman, he advised that an attempt be made to relieve the strangulation under cocaine.

I therefore had the patient removed to St. Joseph's Hospital and with the assistance of Dr. Totman we operated, using one hypodermic syringe full of 1% solution of cocaine. The patient apparently did not suffer any pain. The operation consumed less than 15 minutes, and after cutting the constricting ring we were able to reduce a coil of strangulated gut. The Bassini method for femoral hernia was used to close the wound. She soon ceased to vomit, her pulse improved and before the day was over she begged for her pipe, which I granted to her and she enjoyed two smokes. On the sixth day I permitted her to get up out of bed, and at the end of two weeks she was ready to leave the hospital, but on account of the absence of her son from the city she remained at the hospital two weeks longer. Notwithstanding the extreme age of this patient she still insists on keeping house for herself and son, doing her own housework.

The two points which I wish to make in this brief report are,

First: that a patient can never be too old or too weak to operate for strangulated hernia.

Second: that with cocaine anesthesia much more can be accomplished than is commonly believed.

In last week's Medical Record, John A. Bodine of New York, reports 300 successful cases of hernia operated with cocaine, ten of which were strangulated. However, his oldest patient was only 80 years of age. At the Indiana State Medical Association meeting at Indianapolis in June, 1904, Dr. David C. Peyton of Jeffersonville, Ind., having successfully operated a case of strangulated hernia in a woman 84 years old, read a paper upon the treatment of strangulated hernia in very old persons. In that paper he gave report of the oldest patients suffering from strangulated hernia, from several prominent surgeons to whom he had sent letters of inquiry who had successfully operated. The following is the list of the various surgeons from whom he heard:

Dr. Ransohoff, Cincinnati, patient's age 75.
Dr. B. Merrill, Ricketts, patient's age 76.
Dr. P. S. Conner, Cincinnati, patient's age 83.
Dr. Jos. R. Eastman, Ind., patient's age 71.
Dr. Smythe, Memphis, patient's age 77.
Dr. Edwin Walker, Evansville, patient's age 82.
Dr. John B. Murphy, Chicago, patient's age 66.
Dr. Wm. J. Mayo, Rochester, Minn., 5 patients over 80.
Dr. Cartledge, Louisville, patient's age 75.
Dr. Jno. Young Brown, St. Louis, patient's age 70.
Dr. Wm. B. Coley, New York, patient's age 80. (Cocaine used as anesthetic).
Dr. DeForest Willard, Phila., Pa., operated on a patient 95 years old and the patient lived three years after the operation.
Dr. Oschner, Chicago, patient's age 81.
Dr. Oliver, Indianapolis, patient's age 78.
Dr. M. F. Porter, Fort Wayne, patient's age 70.
Dr. Arthur D. Bevan, Chicago, patient's age 70.

In Syracuse, Dr. John VanDuyne's oldest successful strangulated hernia was 84 years, cocaine used. Dr. Nathan Jacobson operated on a patient 90 years old for strangulated hernia. Dr. D. M. Totman's oldest case was 80 years. Dr. G. M. Price has operated on one 82 and Dr. F. W. Sears on one 72.

507 SOUTH WARREN STREET.

